# Medical students’ participation in the Volunteering Program during the COVID-19 pandemic: a qualitative study about motivation and the development of new competencies

**DOI:** 10.1186/s12909-022-03147-7

**Published:** 2022-02-19

**Authors:** Marina Alves Martins Siqueira, Matheus Belloni Torsani, Gustavo Rosa Gameiro, Lucas Albuquerque Chinelatto, Bruna Chacon Mikahil, Patricia Zen Tempski, Milton A. Martins

**Affiliations:** Center for Development of Medical Education, Faculdade de Medicina, Universidade de São Paulo, Av Dr Arnaldo, 455 Sala 2349, São Paulo, SP ZIP code 01246-903 Brazil

**Keywords:** Volunteering, Medical Students, COVID-19, Motivation, Leadership, Curriculum

## Abstract

**Background:**

Considering evidence on competency-based curricula and the benefits of volunteering, this study highlights innovative ideas to improve medical education during the COVID-19 pandemic. We investigated the motivations and perceptions of competencies developed as leadership and management skills in medical students who joined the COVID-19 Volunteering Program in a Brazilian medical school.

**Methods:**

We performed a cross-sectional, qualitative study involving medical students from the University of São Paulo, Brazil. They were invited to participate in an institutional Volunteering Program during the pandemic and filled out online application forms, including sociodemographic fields and two open-ended questions about their motivation to volunteer and perceptions of their own competencies. At the end of the program, students who were involved in management-related activities were also invited to participate in focus group interviews to track their perceptions about volunteering in this area. Data were submitted to descriptive and content analysis methods. All participants provided informed consent with electronic signatures.

**Results:**

A total of 286 medical students subscribed to the Volunteering Program: 171 (60%) were men, 152 (53%) were enrolled in their 5th year of medical school, and 158 (55%) were 23-25 years old. One hundred and twelve (44%) students reported that they were motivated by altruistic reasons, 95 (37%) reported duty and 47 (19%) prioritized academic interests. Concerning CanMEDS competencies, 91 (36%) students’ responses matched the Scholar component, followed by 51 (20%) with Collaborator, 49 (20%) with Professional, 32 (13%) with Communicator, 17 (7%) with Leader and 11 (4%) with Health Advocate.

In focus groups, students reported the importance of management and leadership skills as a curricular component, motivations to volunteer, and acquired skills from volunteering in management and leadership-related activities, thereby indicating the development of resilient attitudes.

**Conclusions:**

Students who participated in the School of Medicine of University of Sao Paulo (FMUSP) Volunteering Program reported being motivated to help others (altruistic reasons) and to serve society as future health professionals (duty). Knowledge and work-related competencies prevailed over leadership or soft skills, emphasizing the importance of including such activities in the curriculum. Participating in management-related activities could help develop a more resilient attitude toward medical training. Volunteering programs offer students opportunities to develop competencies essential for their roles as future health professionals. Thus, we should think about including such activities in the curricular structure.

**Supplementary Information:**

The online version contains supplementary material available at 10.1186/s12909-022-03147-7.

## Background

The confirmation of the coronavirus disease 2019 (COVID-19) pandemic by the World Health Organization on March 11, 2020, led to the adoption of many measures to decrease the spread of the disease. By the time this article was submitted, according to the World Health Organization (WHO) Coronavirus Dashboard, there have been 220,563,227 confirmed cases of COVID-19, including 4,565,483 deaths. Additionally, a total of 5,289,724,918 vaccine doses had been administered [1]. Precautionary measures to avert the spread of the disease had a profound impact on the global society as a whole and directly affected most businesses and schools, since stay-at-home mandates closed all non-essential enterprises [2, 3]. As essential service providers, health systems faced a double burden of rapidly expanding numbers of patients and imminent resource scarcity [4–8], which required a massive reorganization of services, logistics management and staff. In medical schools, more specifically, while most classes pivoted from in-person to online, volunteer projects emerged to directly or indirectly help the fight against COVID-19 [9], providing students with the opportunity to help medical staff and bringing awareness to their social accountability [10]. At the same time, medical students in their clinical years were highly affected by the significant reduction in areas of practice unrelated to COVID-19 care, and only in the future will we be able to assess its impact [11].

In São Paulo, Brazil, the State Council of Universities decided to suspend both on-campus classes and extracurricular activities in March 2020. The exceptions were health sciences internships [12]. Consequently, faculty had to rapidly reshape activities to the virtual environment, as well as adjust internship rotations [13]. At the School of Medicine of the University of São Paulo, medical students were invited to participate in the Volunteering Program and support health and education services linked to the university. Under the direction of the University Educational Dean, students could opt to volunteer for activities involving health assistance, research, education and hospital administration, according to the required skills and responsibilities. They were advised that time spent on voluntary activities should not pose a hindrance to curricular activities [3].

Volunteering is defined as the dedication of an amount of time to activities without any expectation of compensation. However, there are occasional collateral benefits for those who take part in voluntary services, ranging from physical to psychological effects [14]. According to previous studies involving undergraduate students, the development of leadership, self-confidence, critical thinking and conflict mediation skills are immediate benefits derived from volunteering. Moreover, volunteering is associated with higher commitment to community and social values [15–18]. Students who volunteer are found to be more tolerant and open-minded people, and they are more likely to perform voluntary activities after graduation [16].

While the motivation to volunteer is mainly an individual decision, societal forces have an impact to some degree. A study involving students from universities in six countries—Belgium, Japan, Canada, United States, China and Finland—showed that to younger, economically active individuals, the willingness to volunteer is mostly associated with an opportunity to acquire skills or improve job opportunities, while older, retired volunteers are influenced to a greater degree by altruistic reasons [15].

Another relevant factor is students' motivation in choosing medical careers; according to a literature review, students are motivated mainly by scientific and financial interests, followed by humanistic reasons [17].

To guarantee excellence in medical education and health assistance and prepare physicians who fulfil societal needs and reflect values of the next generation of health professionals, the Canadian Medical Education Directives for Specialists (CanMEDS) from the Royal College of Physicians and Surgeons of Canada proposed the Physician Competency Framework [19]. Competency-based curricula are ultimately necessary for 21st century physicians, as they prioritize accountability, integration with the health system, and learning outcomes and promote student-centered learning [20–22]. Considering this framework, subjects involving the development of leadership skills, such as health systems politics and health services administration, should be given more attention in the medical core curriculum worldwide.

The aim of the present study is to understand what motivated students to volunteer during the COVID-19 outbreak and present competencies they reported developing as a result of their volunteer work, especially those involving management-related activities, which are usually underrepresented in the core curriculum.

## Methods

We opted for organizing methods according to the Consolidated Criteria for Reporting Qualitative Studies (COREQ): a 32-item checklist for interviews and focus groups [23] (see Supplementary Material).

### Research team and reflexivity

The Volunteering Program application form was designed by two of the researchers, M.A.M. S, MD and M.B.T., MD, including sociodemographic profiles and two open-ended questions evaluating the motivation and competencies of applicants to volunteer during the COVID-19 pandemic. Focus groups were conducted online, by M.A.M. S, MD, M.B. T, MD and G.R. G, MD, after the analysis of form responses.

M.A.M. S and M.B.T. were coordinators of the Volunteering Program and medical preceptors at FMUSP. They were responsible for organizing and distributing students among different volunteer initiatives during the COVID-19 pandemic. By that time, they had decided to conduct this original research and document the Volunteering Program and investigate the motivations, competencies and perceptions of the medical students who took part.

M.A.M. S, P.Z.T. and M.A. M had previous experience with a qualitative study design [24, 25].

### Study design

#### Theoretical framework

We performed a cross-sectional, qualitative study based on content analysis of open-ended questions and focus group interviews.

Content analysis involves organizing and eliciting meaning from the data collected to draw realistic conclusions. We performed this method according to Bengtsson’s framework, which includes *decontextualization, contextualization, categorization, and compilation* of data [26].

Open-ended questions were chosen as a first approximation and diagnosis of motivation and competencies of the whole group of medical students enrolled in the Volunteering Program. We considered the risk of obtaining very superficial data and then intended to deepen our understanding by performing focus groups with a smaller convenience sample.

Focus group interviews are an excellent method to understand, in a qualitative study context, not only the general perception of the participants on any subject but also to help researchers identify participants’ perceptions, thoughts, opinions, feelings and nonverbal expressions that go beyond traditional quantitative methods [27, 28].

#### Sampling

The study involved 286 medical students enrolled in the 4th, 5th and 6th years of medical programs who subscribed to the Volunteer Program at the University of São Paulo, Brazil. A convenience sample of seven students was invited to undergo online focus group interviews to explore their perceptions of the volunteer experience.

### Local structure of the Brazilian medical program and participants

In Brazil, a medical degree is obtained in a 6-year undergraduate program, which is traditionally divided into three periods: basic sciences (1st and 2nd years), clinical sciences (3rd and 4th years) and clerkships (5th and 6th years) [29]. Our Volunteer Program included students from the 4th, 5th and 6th years (n=525) who already had clinical experience.

### Data collection

We obtained data from the Volunteering Program application forms, with authorization of the Dean. Data were properly anonymized, transcribed to a separate spreadsheet without the names of applicants by a researcher who was not involved in data analysis. Informed consent was obtained at the time of original data collection [30]. Before focus group interviews, participants also provided informed consent online and with electronic signatures. The research ethics committee of the School of Medicine of the University of São Paulo approved this study.

Open-ended questions, included in the volunteer subscription form, were provided by the authors, who were also coordinators of the Volunteering Program. The questions were as follows:

1. What motivates you to volunteer during the COVID-19 pandemic?
2. Which of your skills and attitudes do you think can best contribute to the volunteer program?

Questioning directions for focus groups were designed according to the Krueger & Casey categories of questions [28]:

1. Which management-related activities have you performed as a volunteer during the COVID-19 pandemic?
2. How would you describe the approach to health-administrative and leadership skills during medical training? Is there any curricular activity involving this subject?
3. What motivated you to choose volunteering for health-administrative activities during the COVID-19 pandemic?
4. What knowledge or skills that you learned through this experience was most important?

Each group included 3 and 4 participants, respectively, and sessions lasted approximately 1 hour. Each student participated only once. The interviews were guided by M.A.M. S. M.B. T and G.R.G. were also present to take notes for further discussion. Participants were provided with information about the study objectives, methodology and confidentiality at the beginning of all sessions. Questions were open-ended, carefully thought out to promote interaction, discussion, and reflection and to explore participants' perspectives and criticisms about volunteering. All interviews were performed through online conferences, recorded with participants’ consent and transcribed for analysis. No pilots or repeated interviews were conducted.

### Data analysis

Responses to the open-ended questions were available on an anonymized spreadsheet. Focus groups were recorded in video and transcribed by professional online service providers. There was no return to participants for comments and/or correction of transcriptions.

We adopted content analysis methods [26, 31–37] for the analysis of responses to the open-ended questions and focus group interviews. The researchers started with a free reading of the transcribed text, without the intention of categorization. During the second reading, they proceeded to the categorization of emerging themes and derived issues separately, until saturation of data. Finally, each researcher’s products were paired with similarities in meaning and were discussed with the research group. The results were divided into analytical categories and examples. Some examples were selected to illustrate each of the elected categories and were translated into English.

### CanMEDS 2015 Physician Competency Framework

The only pre-existing categorization used in this study was the Canadian Medical Education Directives for Specialists (CanMEDS) from the Royal College of Physicians and Surgeons of Canada [19] to guide the analysis and to categorize the students’ answers about their competencies. According to this framework, competencies for future physicians can be categorized and described as follows:

Communicator: forms relationships with patients and their families that facilitate gathering and sharing of information. The professional must adopt active listening techniques and an empathic, patient-centered approach. He or she must share decisions and guarantee patient privacy and information confidentiality.

Collaborator: works effectively with other health professionals, providing safe, high-quality, patient-centered care.

Leader—contributes to the vision of a high-quality health care system, provides excellent patient care as clinician, administrator, scholar or teacher.

Health advocate—employs his or her expertise and influence on behalf of the population needs, supports the mobilization of resources to effect change.

Scholar—demonstrates a lifelong commitment to excellence in practice through continuous learning. Teaches and supervises new learners and evaluates and produces scientific evidence.

Professional—commits to his or her patients and population health and well-being through ethical practice, accountability and maintenance of personal health.

### Descriptive statistics

In addition to qualitative analysis, we assessed some demographic variables, such as gender, age, year of medical program, pre-existing diseases included in risk groups for COVID-19, type of transportation used, and interest in six areas of voluntary activities. We used descriptive statistics to analyze demographic data using SPSS Statistics for Windows, Version 22.0 (released 2013, IBM Corp, Armonk, NY).

## Results

### Volunteers’ sociodemographic profile

Of 525 students enrolled in the 4th, 5th and 6 h years of medical training, 286 (54.4%) applied to our COVID-19 Volunteering Program. Thirty-five applications (12.2%) were considered missing values; after we identified blank fields in the subscription form, we could not include them in any category for qualitative analysis.

Regarding sample characteristics, 171 (60%) were men, 152 (53%) were enrolled in the 5th year of medical school, 158 (55%) were 23 to 25 years old, and 111 (39%) reported they walked to volunteer sites (Table 1).

**Table 1:** Volunteers demographic distribution

Variables		Number of students*
Gender	Female	115 (40%)
	Male	171 (60%)
	Other	0 (0,00%)
Year in medical program	4^th^	83 (29%)
	5^th^	152 (53%)
	6^th^	51 (18%)
Age group	20-22 years old	73 (26%)
	23-25 years old	158 (55%)
	≥ 26 years old	55 (19%)
Risk group	Yes	3
	No	283
Transportation	Public transport	62 (22%)
	Private car	103 (36%)
	App services	7 (2%)
	Bike/Motorcycle	2 (1%)
	Walking	111 (39%)

*Percentages were calculated according to the number of Volunteer Program subscription forms (286 students)

The COVID-19 Volunteering Program application form allowed students to choose between six areas of interest: (1) COVID-19 patient clinical assistance; (2) non-COVID-19 clinical assistance; (3) epidemiological surveillance; (4) clinical research; (5) support for online educational activities; and (6) hospital administration (Table 2).

**Table 2:** Student distribution according to areas of interest

Areas of interest**	COVID-19 patients clinical assistance	202 (26%)
	Non-COVID-19 clinical assistance	230 (30%)
	Epidemiological surveillance	102 (13%)
	Clinical research	102 (13%)
	Support of online educational activities	69 (9%)
	Hospital administration	74 (9%)

**Total percentage exceeded 100% because it was possible to choose more than one option in this section

### Open-ended questions: Motivation to volunteer

Concerning students’ motivations to volunteer, some themes emerged from their responses, as follows (Table 3):

1. Altruistic reasons
  Since the COVID-19 pandemic started, medical students promptly offered their help as volunteers. They reported a great willingness to contribute to decreasing the harm caused by the disease, from assisting patients to helping professional colleagues, without expectation of any reward.
  *“I believe we are going through a unique and delicate moment of great difficulty. I would like to help and try to minimize the impact of the COVID-19 pandemic on patients and health care professionals”* (G.P.M.E. Male, 22 years old)*.*
  *“What motivates me is to help people who are going through great suffering. I would like to help my health care colleagues who are working and exposing themselves to take care of all the patients. I am willing to do whatever work is needed”* (G.L. Female, 22 years old)*.*
  Some of them also left their homes to get closer to the hospital and protect their loved ones from infection in case they were recruited to help in the health services.
  *“I decided to volunteer for the same reasons that I have chosen the medical career. I am living far from my parents, who are from risk groups for COVID-19 infection, which makes me more available to help. I wish I could contribute, making health assistance as efficient as possible and diminishing health professionals’ workloads. Regarding my limitations as a student, I am willing to help whenever it is necessary”* (C.D. Female, 23 years old)*.*
2. Duty
  Another group of students reported that they felt socially responsible for helping in the pandemic as future health professionals. Additionally, being enrolled in a medical course at a public university was strongly associated with a sense of debt toward society.
  *“Since I study in a public university, I believe I have a social obligation in the face of such a critical moment as the COVID-19 pandemic”* (G.C. Male, 24 years old)*.*
  *“The critical circumstances imposed by the COVID-19 pandemic and the challenges it brings for our health system make me feel responsible for helping to fight against the disease”* (C.O. Male, 24 years old)*.*
  *“I believe medical students have taken a great pledge to assist the population as future doctors graduating from a public university.... I also volunteered as a way to confront the feeling of powerlessness I believe a lot of my colleagues are facing too”* (G.T. Female, 24 years old)*.*
3. Academic interests
  Some students reported being attracted by the opportunity to actively join the effort to control the pandemic as volunteers, since they identified this as a unique learning experience. They were interested in learning about clinical aspects of the infection and understanding the health system organization in the face of this peculiar situation.
  *“We are under a state of emergency, a pandemic with proportions the world has not faced in a hundred years. I am proud of my university and hospital for being an example for the rest of Brazil. I think the learning experience we can take out of it is huge. Medicine is the career I chose for my life, and helping is a way of thanking all of my colleagues currently working on the frontline against COVID-19”* (L.B. Male, 22 years old)*.*
  *“I would like to help in the effort against the pandemic and to put all the knowledge acquired during my undergraduate program into service of the population. I am also interested in learning how to manage COVID-19 cases and how the health system is organizing in the midst of this unprecedented global shift”* (G.M. Male, 21 years old)*.*
  *“As an intern, I believe I am able to help with some essential tasks during the pandemic. Moments such as this are essential for our education as health professionals. In addition, we're in a great public university, and this volunteer opportunity would be a way of giving back all it has provided me”* (A.L.B. Female, 22 years old)*.*
  *“I have great interest in emergency medicine, and I intend to apply for residency in this area. Therefore, in the pandemic context, in addition to assisting patients, I could also learn more about this specialty. I believe the volunteers will help to unburden our health system and allow doctors to treat the patients with more time and quality, while we help with minor tasks”* (G.B.C. Female, 24 years old)*.*

**Table 3:** Categories and issues for the theme: “Motivations to volunteer during the COVID-19 pandemic”

Category	Issues	Examples
Altruistic reasons	Assisting patients	“Help people who are going through great suffering.”
	Helping health professionals	“Help my health care colleagues who are working and exposing themselves.”
	Willingness to help	“I am willing to help whenever it is necessary.”
Duty	Studying at a public university	“Medical students take a great pledge to assist the population as future doctors, graduating from a public university.”
	Moral obligation as future health professionals	“Feel responsible for helping to fight against the disease.”
Academic interests	Learning about a new disease	“Learning how to manage COVID-19 cases and how the health system is organizing in the midst of this unprecedented global shift.”
	Applying previous knowledge	“Puting all the knowledge acquired during undergraduate education into serving the population.”

**Fig. 1:**
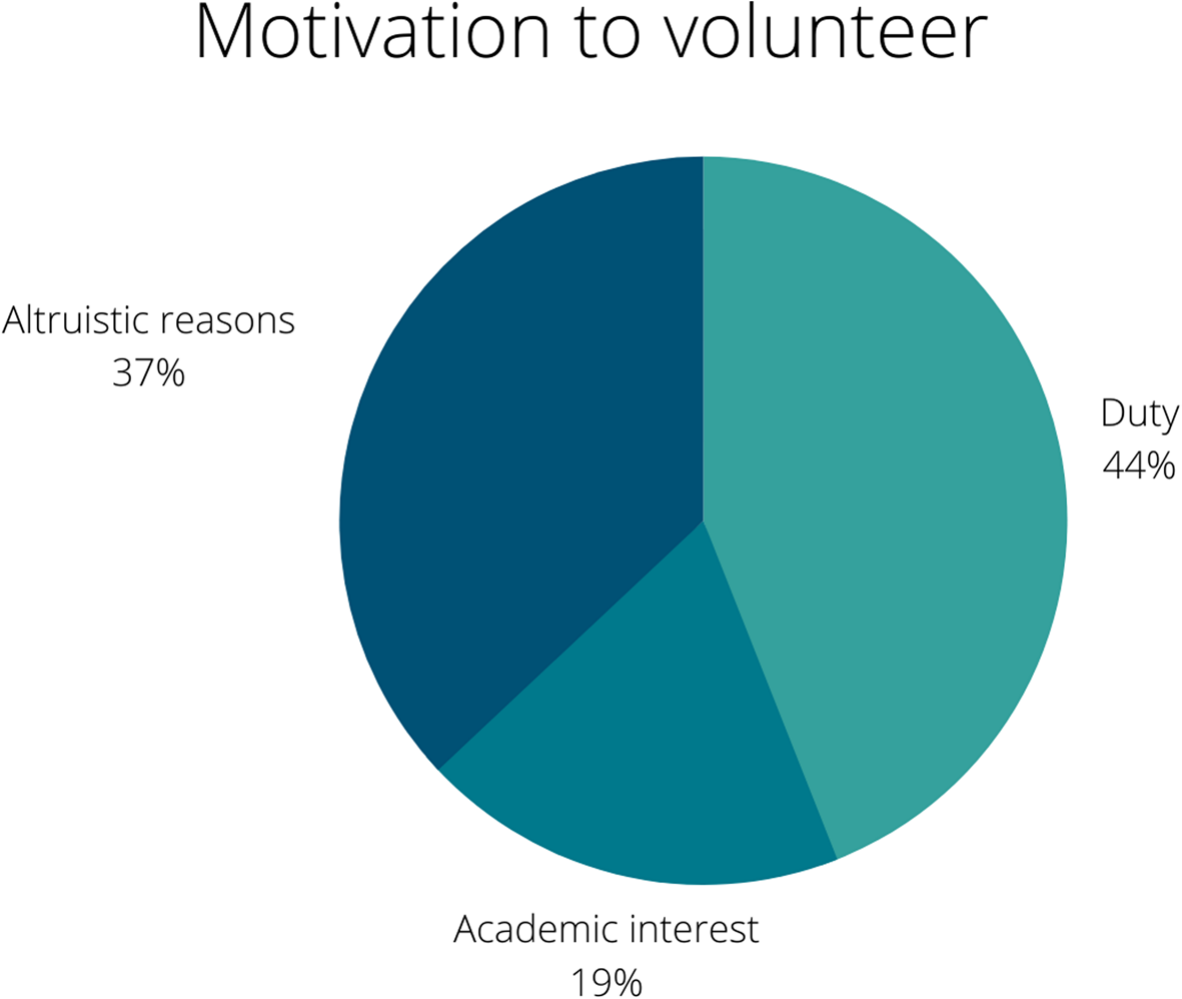
Motivation to volunteer

Most of the responses referred to altruistic reasons to volunteer (44%), followed by duty (37%) and academic interests (19%) (Fig. 1).

### Open-ended questions: Volunteers’ perceptions of their competencies

Regarding students' perceptions of their own skills, we performed content analysis of responses based on the six competencies framework from the Royal College of Physicians and Surgeons of Canada (CanMEDS, 2015) (Table 4):

1. Communicator
  Students who reported abilities associated with establishing relationships and providing information and orientation to patients and families were categorized as communicators, their outstanding competency, as in the following example:
  *“I am communicative, very easy-going and helpful. I wish I could participate in activities involving patient orientation about COVID-19 or assisting inpatients in wards (COVID-19 or not). I'd really enjoy working in those areas”* (G.D. Male, 23 years old)*.*
  *“I believe that interpersonal relationships and communication skills are some of my strengths, so I could help in patient care. Furthermore, I am dedicated to learning and keep myself updated about the current situation. It might be of great help in screening and assisting patients. I also have experience in volunteer projects”* (L.M.S. Male, 25 years old)*.*
2. Collaborator
  Students who mentioned collaborative skills defined themselves as proactive and helpful. They were willing to learn new things to contribute to the work team and improve patient care.
  *“I am willing to work wherever I can be helpful! I am committed to learning any necessary skill and giving all I can to help my colleagues and patients throughout this pandemic. One could say that I am a committed, organized and hardworking person. I am also willing to develop new competencies to help in any kind of volunteer activity”* (A.R. Female, 24 years old)*.*
3. Leader
  Leadership could be identified in students who reported experiences with student representation on university councils or were enrolled in extracurricular activities, generally performing administrative tasks. We observed that this group was also predominantly motivated to volunteer due to social duty awareness.
  *“I believe that I am able to lead and organize people in a team, which might be useful in many contexts. I have worked on a research project during an exchange program for one semester, and I have developed some ability in this area. I have been class representative for many semesters, and therefore, I have learned a little about students' demands related to online education, which could be useful in volunteer activities. I am only in the 4th year, so although my medical abilities are limited, I am very interested in medicine and a keen learner”* (A.B. Female, 22 years old)*.*
4. Health advocate
  The health advocate profile was the least common among students. This group of students showed higher awareness about the importance of public measures to contain the spread of COVID-19.
  *“I am a communicative, collaborative, resilient person. I also like to work in a team. I would like to perform activities involving patient assistance or epidemiological data assessment. I think it is very important to help the population raise awareness about the COVID-19 pandemic to control it”* (N.R. Female, 24 years old)*.*
  *“I really enjoy forming a relationship with patients. I am also a proactive person, and I understand the relevance of every step in a workflow. Therefore, I am available to help in any task that involves patient care. I am a senior, and I have already done the emergency rotations (cardiology, clinic, surgery and neurology), which gave me good theoretical and practical knowledge. Furthermore, I am totally motivated to help our hospital through this period. I have even left home and moved closer to the hospital in order to avoid infecting my loved ones. I am at your service”* (A.A.M. Male, 25 years old).
5. Scholar
  A considerable number of students described themselves as academically prepared to take part in voluntary activities. They mentioned previous knowledge and academic experiences as potential contributors to the Volunteering Program.
  *“I have some knowledge from previous experience in non-COVID internships (Neonatology and Obstetrics) and a great interest in internal medicine and clinical reasoning, focused on treating COVID patients. I also have previous experience in scientific research (Harvard Exchange Program)”* (A.B. Male, 23 years old)*.*
  *“I am very interested in scientific discussions, and I am currently keeping track of COVID-19 scientific developments. Therefore, I think I would be of great value in research projects”* (P.F.M. Male, 25 years old)*.*
6. Professional
  Students who defined themselves as reliable and ethical called attention to their professional competencies. They commonly reported commitment and seriousness as their main qualities, as follows:
  *“I am very helpful and committed to tasks when they are assigned to me. I am also very willing to learn new things and work in teams. I also believe that my attitude of taking things seriously and not panicking in the face of negative outcomes, always helping my colleagues with their needs and challenges, are good characteristics for volunteer services”* (N.C. Female, 24 years old)*.*
  *“I am very pragmatic and strict with safety guidelines and protocols. I obey orders and question them only at appropriate moments. I am very proactive and capable of making quick decisions. I communicate well with patients in different age groups and look for a broad vision of care.*

**Table 4:** Categories and issues for the theme: “Volunteers’ perceptions of their competencies to volunteer during the COVID-19 pandemic”

Category	Issues	Examples
Communicator	Establishing doctor–patient relationships	“I believe that interpersonal relationships and communication skills are some of my strengths.”
	Patient orientation	“I am communicative, very easy-going and helpful. I wish I could participate in activities involving patient orientation about COVID-19.”
Collaborator	Proactivity and helpfulness	“I am committed to learn any necessary skill and give all I can.”
	Self-updating on behalf of the team	“I am also willing to develop new competencies to help in any kind of volunteer activity.”
Leader	Previous experience in leadership positions	“I have been class representative for many semesters.”
Health advocate	Importance of public awareness	“It is very important to help the population by raising awareness about the COVID-19 pandemic in order to control it.”
Scholar	Previous knowledge and academic experience	“I have some knowledge from previous experience in internships.... I also have previous experience in scientific research.”
Professional	Reliability	“I am very helpful and committed to tasks when they are attributed to me.”
		“I obey orders and question them only at appropriate moments.”
	Resilience	“Taking things seriously and not panicking in face of negative outcomes.”

**Fig. 2:**
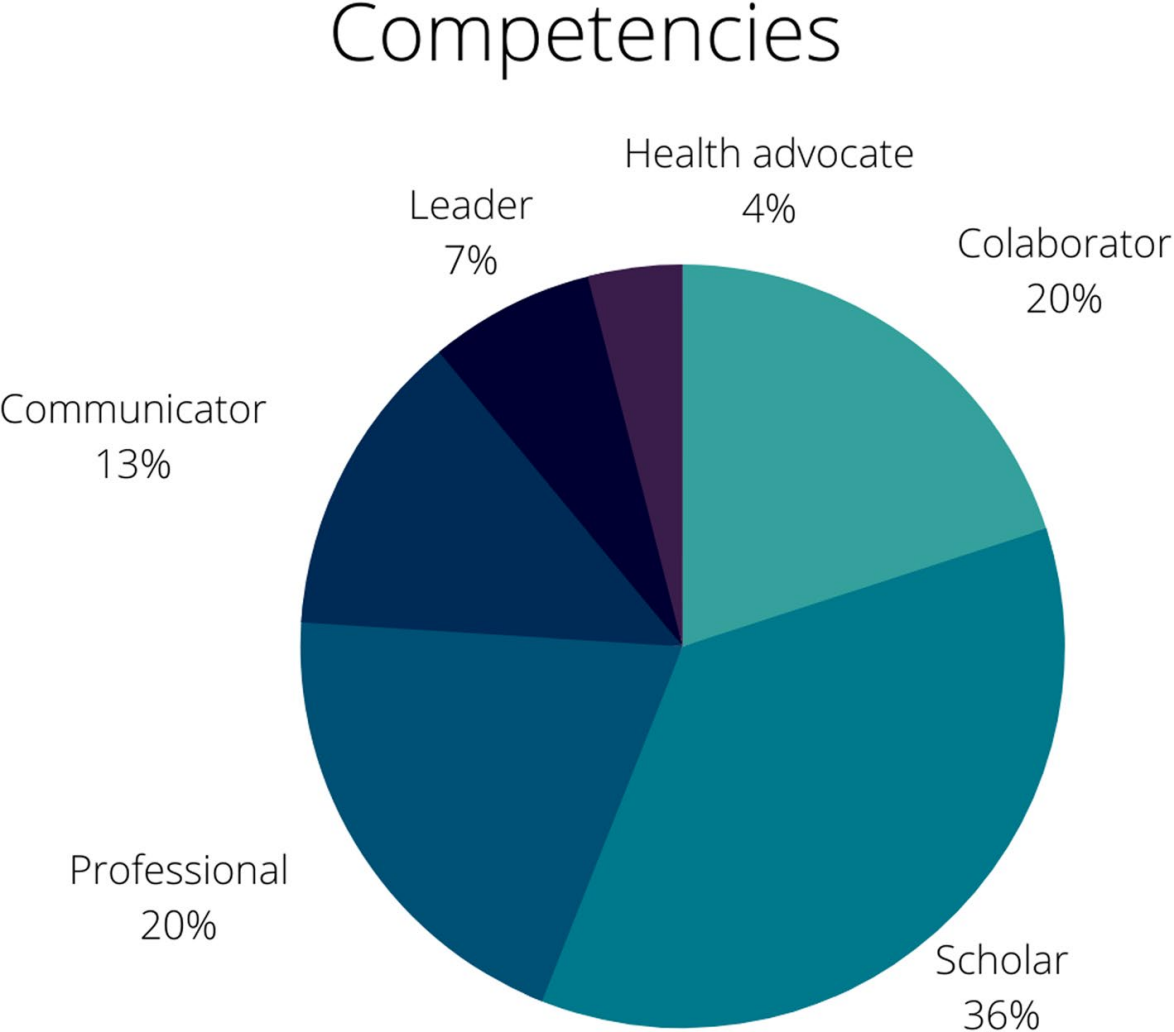
Medical students reported competencies according to CanMEDS

Regarding quantification of responses, most students defined themselves as Scholars (36%), followed by Professionals (20%), Collaborators (20%), Communicators (13%), Leaders (7%) and Health Advocates (4%) (Fig. 2).

### Focus group interviews: Perceptions of management-related activities and the development of leadership skills

The focus group interviews included seven students who participated in health-administrative activities as part of the Volunteering Program. Their discussion was organized into 4 categories and divided into 15 issues (Table 5).

1. Activities performed as volunteers
  Students started the discussion by recalling some experiences they had during volunteering. They highlighted activities such as remote monitoring and providing comfort to patients with a confirmed diagnosis of COVID-19, which was a gratifying experience. They also reported managing a great deal of epidemiological data, which required skills such as teamwork, leadership and organization, as well as technical knowledge to critically analyze evidence about the COVID-19 pandemic. Participating in management-related activities contributed to their development of a sense of social responsibility, autonomy and innovation.
  - Healthcare-related activities
  *“Many times, people who lived alone were scared in the beginning of the pandemic… they didn’t know how their disease would progress… so, we called, and we kept calling, and sometimes from one day to another we recognized the voices… it was gratifying to do this follow up”* (C.H.P*.* Male, 23 years old).
  b. Leadership and innovation
  *“I thought that it was an enriching experience because we worked with different people. Furthermore, we managed to work in a group to organize that huge amount of data and try to come up with something productive from it”* (M.B. Male, 20 years old).
  *“I think [writing the institutional COVID-19 protocol] was something very authoral, as it was our idea. [The supervisors] were just guiding us. We had weekly meetings with them and some among ourselves. After that, we decided how to write. We divided the work, and each one wrote on a topic”* (R.C.M. Male, 25 years old)*.*
  *“What I found interesting was precisely the fact that none of us knew how to write this document. So, everything was being built, and the supervisors experience as managers helped us a lot to see what would be feasible and applicable in our reality”* (D.M. Female, 25 years old)*.*
  *“In fact, besides thinking about new ideas, we have adapted many things to the reality of the University of São Paulo. Whether we wanted to or not, we discussed it a lot (...) and we couldn’t find almost anything in other documents”* (R.C.M. Male, 25 years old)*.*
  iii. Research
  *“It was a secure experience because we talked by phone, we received the orientations, we were prepared… We could communicate with people and learn from them how the symptomatology presented… I think that in this aspect we learned and discovered the disease”* (F.R.S. Male, 27 years old)*.*
  *“First, we conducted research to develop the basis of the USP COVID-19 protocol… knowing the evidence about social distancing, masks, PPIs, and tests…”* (M.C.P. Female, 24 years old)*.*
2. Importance of management and leadership skills as a curricular component

**Table 5:** Data from focus group interviews: Students’ perceptions of management-related activities and leadership skills developed during the volunteer program and in medical training

Category	Issues	Examples
Recalling experiences	Healthcare-related activities	“It was gratifying to do this follow up.”
	Leadership and innovation	“We managed to work in a group to organize that huge amount of data and try to come up with something productive from it.”
	Research	“Knowing the evidence about social distancing, masks, PPIs, tests and trying to bring that to USP reality.”
Perceptions over curriculum enhancement	Private practice	“You will occasionally have a management position in your private practice.”
	Public-health	“Our university is public, and after our graduation, we will work fulltime in the Public Health System.”
	Reflection on action	“I felt that our teachers took very little responsibility for discussing this with us.”
Motivations to volunteer	Exploring new opportunities	“It could be an opportunity to learn something new that I would not learn during graduate school.”
	Altruistic reasons	“Contributing in some way to help in this moment of pandemic.”
	Academic interests	“I need to apply things, and that is the way I learn best.”
Acquired skills	Resilience	“Dealing with the frustration of an uncontrolled environment.”
	Collaborator	“Being more comprehensive with the limitations.”
	Communicator	“What touched me the most in this voluntary process of vigilance was the importance of communication.”
	Professional	“We felt we understood what the difficulties were and built solutions to meet those challenges.”
	Scholar	“There was some technical learning involved, for example: I had never performed a literature review before.”

It was unanimous among students that there is an ultimate need to include management and leadership in the medical curriculum. However, students differed on the focus of teaching. Some of them considered these skills important mainly for private practice in the future, but others thought that learning about public-health administration was fundamental and, coming from a public school, this was quite a moral obligation. They also expressed the desire for a curricular activity in which they could discuss political and administrative issues that affect their academic experience as future health professionals.

- Private practice

*“... you will occasionally have some management position in your private practice, in a hospital, in the health system, public, private… even in the micro, if you have a small room as your office, you already have to do some management of supplies, human resources, logistics… and, in that sense, we have zero knowledge”* (J.P.T. Male, 23 years old)*.*

*“In a hospital, public or private, having management skills is very important, and it ends up that, in Brazil, these positions used to be occupied by nonhealth professionals. However, we see that has started to change recently, [as many doctors are starting to get trained for this]. I really don't know if there is any medical school that includes [management or leadership skills] in the curriculum, but I think it is very necessary. It is also important for private practice in the future. We leave college without knowing how to manage our own clinics”* (R.C.M. Male, 25 years old)*.*

b. Public health

*“It is interesting to see in the macro view, you know? How the hospital was in that moment. We had a good idea of how the hospital was affected during the worst of the pandemic… It was very interesting to see from that point of view”* (C.H.P*.* Male, 23 years old)*.*

*“In fact, there should be room for management and leadership at graduation, especially in terms of public health. Because our university is public and until we graduate, we will work in the Public Health System”* (D.M. Female, 25 years old)*.*

iii. Reflection on action

*“I think that our teaching hospitals, for example, provide be a good opportunity to include management in the undergraduate program. We went through several crises and are still going through some, in terms of hiring issues and lack of personnel, excessive demand, participation of private initiatives and so on. There are so many contradictions in terms of management and in terms of public health, but I feel that our teachers take very little responsibility for discussing this with us”* (M.C.P. Female, 24 years old)*.*

(3) Motivation to volunteer

The sample of students invited to participate in focus group interviews showed very similar motivations to volunteer. They reported altruistic reasons, academic interests and exploring new opportunities.

- Exploring new opportunities

*“I have chosen volunteering in management because I thought it could be an opportunity to learn something new that I would not learn in school. Although I felt unprepared at the time, I think it was a great experience”* (D.M. Female, 25 years old)*.*

*“... In the beginning, I was afraid of the administrative field, so it was not my first option for volunteering. I have never felt capable, maybe because during school we do not have many experiences like that.... Despite that, the volunteering program was a great opportunity to take the risk and overcome fear”* (D.M. Female, 25 years old)*.*

b. Altruistic reasons

*“… The idea of contributing in some way to help in this moment was of interest to me. As I was far away from my family and could be available… I wanted to help...”* (C.H.P*.* Male, 23 years old)*.*

*“I believe that we, as future doctors, need to develop a sense of leadership and responsibility, and the fact that I study in a public university makes me feel like I have a moral obligation to return to society what was invested in me”* (D.M. Female, 25 years old)*.*

iii. Academic interests

*“I applied for almost every project at the Volunteering Program because I did not want to stay at home during the pandemic.... I find it very difficult to learn by reading books or attending theoretical classes. I need to apply things, and that is the way I learn best”* (M.C.P. Female, 24 years old)*.*

(4) Acquired skills from volunteering in management and leadership-related activities
  This session also found issues similar to those revealed by the volunteers’ responses in "Open-ended questions: Volunteers’ perceptions on their competencies". Students discussed the same CanMEDS competencies such as the Collaborator, the Communicator, Scholar and Professional. However, a new issue came up, which was Resilience. Students reported developing an ability to cope with frustrating situations, such as characteristic delays on referrals in the health system, and they empathized with professionals as they watched closely the work involved in managing such issues. Here are some examples to better understand this process:
  - Resilience
  *“From the moment we jumped into this management thing, we learned to deal with the frustration of an uncontrolled environment, not controlled by ourselves or our team”* (M.C.P. Female, 24 years old)*.*
  b. Collaborator
  *“… because we are used to only criticizing the public administration and the way things are done especially in critical situations where nobody has answers, understanding and living the limitations… made people sympathize with others who are in those administrative positions and better comprehend the limitations…”* (J.P.T. Male, 23 years old)*.*
  iii. Communicator
  *“I think that what I will carry with me, what touched me the most in this voluntary process working in epidemiological surveillance was the importance of communication”* (C.H.P*.* Male, 23 years old)*.*
  iv. Professional
  *“… it showed the necessity of organization, management of people, like the colleagues that were also participating in the epidemiological surveillance... It was great work that we did together. We understood what the difficulties were and built solutions to meet those challenges”* (F.R.S. Male, 27 years old).
  e. Scholar
  *“I think there was some technical learning involved. For example, I had never performed a literature review before or all the work of choosing and reading articles, to create a large group of references” (*M.C.P. Female, 24 years old)*.*

## Discussion

Of the 286 medical students who applied to the Volunteering Program, the majority were men, in the 5th year of medical school and with ages 23 to 25. Students who volunteered in the FMUSP Volunteering Program had a high prevalence of self-perceived scholarly, professional and collaborator profiles. Health advocate was the least represented competency. This underrepresentation is probably due to the low frequency of this competency in the curriculum [38] and due to a low appraisal by students [39]. Altruistic reasons and duty prevailed as motivations to volunteer. In focus groups, students reported the importance of management and leadership skills as a curricular component. They also discussed their motivations to volunteer, the skills they acquired from volunteering in management and leadership-related activities, including the development of resilience.

Resilience may be associated with students' perception of keeping themselves actively working, even in anxiogenic and burnout-propitious moments such as during the COVID-19 pandemic [40]. Resilience might also be related to engagement in nonmedical extracurricular activities [41].

These findings provide a glimpse of areas in which policy-makers should invest in qualified medical education, including programs of faculty development and constructing a competency-based curriculum. Faculty should also encourage social volunteer projects, as they seem to increase soft skills and resilience. This may result in a higher quality of life for medical students and improve their perceptions of the educational environment [29].

Concerning motives to volunteer, altruistic reasons can be associated with the acute crisis imposed on public welfare services [15] in the context of the pandemic that victimized millions of people and defied health systems globally [1, 13].

According to the literature, interests in volunteering may also be related to the construction of professional identity. Most of the students chose to work in patient care, which can be considered an effort to develop their identities as doctors through “apprenticeship” [42]. In addition, it might represent an opportunity for students to develop a sense of peer unity as medical students and within medical schools [42, 43].

Having the opportunity to participate in actions against the pandemic, while others must stay at home isolating themselves, gave the student a sense of purpose and social responsibility. This may increase their interest in volunteering throughout this period. In addition, previous studies suggest that social interactions play an important role in willingness to volunteer, which is more significant than the activity itself [44].

It is important to note that students must develop a pool of competencies throughout medical training, and identifying their perceptions about their own skills is valuable data for faculty to reinforce curricular and institutional strengths and mitigate deficits. Students have mostly reported knowledge and work-related competencies rather than those associated with leadership or soft skills. These findings agree with those of previous studies, which report that the career choice of Brazilian medical students is associated with intellectual curiosity and professional autonomy [43–45]. A traditional curricular structure seems likewise to have some influence in these results, as theoretical strategies, poorly focused on learning outcomes, are still expected to prevail [20]. Furthermore, we cannot disregard the hidden curriculum and role modeling influence, considering the academic and research tradition of our institution and faculty [46].

Students' initiatives to volunteer during the COVID-19 pandemic were not restricted to Brazil. In many countries, such as the United States, United Kingdom and Portugal, students and faculty have organized similar programs [47–49]. We believe that these programs will persist after the pandemic, offering students notable professional and personal benefits [14–16, 18].

### New perspectives: Incorporating management and leadership skills in medical curriculum

In view of the positive results that we observed within the volunteer project in management at the HU-USP, our teaching hospital, a proposal was made to reformulate the grade of the medical course in the 6th year. From the experience reported by the students, it was understood to be important to have an internship in hospital management so that the student's training experience was more complete.

Thus, an internship proposal was prepared, which began with a theoretical deepening on the theme, especially focused on the area of ​​regulation of health services, followed by practical training in the medical course workshop for skills development in this area. Finally, in the longitudinal stages in the HU-USP, students will be distributed on specific emergency coordination shifts, together with hospital managers, to develop and evaluate the necessary attitudes for health management competence.

In addition to the elaboration of activities, a framework was also designed to assess students that reaches the four levels of Kirkpatrick's taxonomy [50]: assessment of satisfaction through an assessment form by students after theoretical-practical training; learning assessment with a theoretical test on hospital management and regulation; behavioral assessment through observation by supervisors during practical activity; and an impact assessment that analyzes the performance metrics in regulation performed by students.

### Limitations

The authors acknowledge some limitations in the present study. The students volunteered at their convenience to participate in the focus groups, which may have led to biases. Moreover, despite the efforts of the moderators to remain impartial, they may have influenced the participants, as some of them were the coordinators of the Volunteering Program. In a focus group, furthermore, more participative students might have their opinions emphasized to the detriment of others. Finally, this study was conducted in only one medical school with a limited number of participants in a specific situation (during the COVID-19 pandemic); thus, the external validity needs further research.

## Conclusion

The majority of students reported being motivated to volunteer in the COVID-19 pandemic, to help others (altruistic reasons) and to serve society as future health professionals (duty). These results were expected, considering the impact of the pandemic on the health system and society worldwide. Concerning volunteer competencies, knowledge and work-related competencies prevailed over leadership or soft skills. This emphasizes the importance of developing a more competency-based curriculum and including such activities in the core curriculum. Participating in management-related activities could help develop a more resilient attitude toward medical training.

## Supplementary Information

**Additional file 1.**

## Data Availability

The datasets used and/or analyzed during the current study are available from the corresponding author upon reasonable request. As access to datasets was authorized by the Dean (see on “Methods – Data collection”), authors have chosen to keep datasets publicly unavailable in accordance with FMUSP institutional guidelines and Dean’s Office recommendations to keep participants anonymity.

## Abbreviations

USP: University of São Paulo
FMUSP: School of Medicine of the University of São Paulo
CanMEDS: Canadian Medical Education Directives for Specialists
HU-USP: USP’s Teaching Hospital

## References

[1] WHO (COVID-19) Homepage. WHO Health Emergency Dashboard. Available from: https://covid19.who.int/. Accessed 6 Sept 2021.

[2] Rose S. Medical Student Education in the Time of COVID-19. JAMA. 2020; 10.1001/jama.2020.5227. 10.1001/jama.2020.5227 32232420

[3] Whelan A, Prescott J, Young G, Catanese VM. Interim guidance on medical students’ participation in direct patient contact activities: principles and guidelines. Association of American Medical Colleges. Available from: https://lcme.org/wp-content/uploads/filebase/March-30-2020-Interim-Guidance-on-Medical-Students-Participation-in-Direct-Patient-Contact-Activities.pdf. Access on March 30th, 2020;

[4] Armocida B, et al. The Italian health system and the COVID-19 challenge. The Lancet. 2020;5 Published Online March 25, 2020. DOI: 10.1016/S2468-2667(20)30074-8. 10.1016/S2468-2667(20)30074-8 PMC7104094 32220653

[5] Legido-Quigley H, et al. The resilience of the Spanish health system against the COVID-19 pandemic. The Lancet. 2020;5 Published Online March 18, 2020. DOI: 10.1016/S2468-2667(20)30060-8. 10.1016/S2468-2667(20)30060-8 PMC7104264 32199083

[6] Adams JG, Walls RM (2020). Supporting the Health Care Workforce During the COVID-19 Global Epidemic. JAMA..

[7] Legido-Quigley H, et al. Are high-performing health systems resilient against the COVID-19 epidemic. The Lancet. 2020. Published Online March 6, 2020. DOI;395 10.1016/S0140-6736(20)30551-1. 10.1016/S0140-6736(20)30551-1 PMC7124523 32151326

[8] Croda J, et al. COVID-19 in Brazil: advantages of a socialized unified health system and preparation to contain cases. Rev. Soc. Bras. Med. Trop. 2020;53 Uberaba 2020 Epub Apr 17 10.1590/0037-8682-0167-2020. 10.1590/0037-8682-0167-2020 PMC7182282 32320998

[9] Soled D, Goel S, Barry D, Erfani P, Joseph N, Kochis M (2020). Medical student mobilization during a crisis: Lessons from a COVID-19 medical student response team. Acad Med..

[10] Yang D-Y, Cheng S-Y, Wang S-Z, Wang J-S, Kuang M, Wang T-H (2020). Preparedness of medical education in China: Lessons from the COVID-19 outbreak. Med Teach..

[11] Ahmed H, Allaf M, Elghazaly H. COVID-19 and medical education. Lancet Infect Dis. 2020 Jul;20(7):777–8. ; Mian A, Khan S. Medical education during pandemics: a UK perspective. BMC Med. 2020 Apr 9;18(1):100; 10.1186/s12916-020-01577-y PMC7141929 32268900

[12] Knobel, M. Cruesp divulga comunicado sobre suspensão das aulas a partir de 17/03. Jornal da USP. Campinas, [cited 2020 Mar 13]. Available from: https://jornal.usp.br/institucional/cruesp-divulga-comunicado-sobre-suspenSão-das-aulas-nas-universidades-a-partir-de-17-03/;

[13] Chinelatto LA (2020). What You Gain and What You Lose in COVID-19: Perception of Medical Students on their Education. CLINICS.

[14] Corporation for National and Community Service, Office of Research and Policy Development. The Health Benefits of Volunteering: A Review of Recent Research, Washington, DC 2007;

[15] Hustinx L (2010). Social and Cultural Origins of Motivations to Volunteer A Comparison of University Students in Six Countries. International Sociology..

[16] Sax LJ, Astin AW, Avalos J, “Long-Term Effects of Volunteerism During the Undergraduate Years” (1999). Higher Education. Paper 88.

[17] Goel S (2018). What motivates medical students to select medical studies: a systematic literature review. BMC Medical Education.

[18] Güntert ST, Strubel IT, Kals E, Wehner T (2016). The quality of volunteers’ motives: Integrating the functional approach and self-determination theory. The Journal of Social Psychology.

[19] Frank JR, Snell L, Sherbino J (2015). CanMEDS 2015 Physician Competency Framework.

[20] Frank JR, Snell LS, Ten Cate O, Holmboe ES, Carraccio C, Swing SR, Harris P, Glasgow NJ, Campbell C, Dath D, Harden RM, Iobst W, Long DM, Mungroo R, Richardson DL, Sherbino J, Silver I, Taber S, Talbot M, Harris KA (2010). Competency-based medical education: theory to practice. Medical Teacher.

[21] Brennan T, et al. Medical Professionalism in the New Millennium: A Physician Charter. Annals of Internal Medicine. 2002;136(3). 10.7326/0003-4819-136-3-200202050-00012 11827500

[22] Frenk J (2010). Health professionals for a new century: transforming education to strengthen health systems in an interdependent world. Lancet.

[23] A. Tong et al. Consolidated criteria for reportingqualitative research (COREQ): a 32-itemchecklist for interviews and focus groups. International Journal for Quality in Health Care; Volume 19, Number 6: pp. 349–357. Advance Access Publication: 14 September 2007. 10.1093/intqhc/mzm042 10.1093/intqhc/mzm042 17872937

[24] Siqueira MAM, Gonçalves JP, Mendonça VS, Kobayasi R, Arantes-Costa FM, Tempski PZ, et al. Relationship between metacognitive awareness and motivation to learn in medical students. BMC Med Educ 2020 [cited Dec 15,2020];20.Available from https://www.ncbi.nlm.nih.gov/pmc/articles/PMC7602298/; 10.1186/s12909-020-02318-8 PMC7602298 33126882

[25] Tempski P, Bellodi PL, Paro HB, Enns SC, Martins MA, Schraiber LB (2012). What do medical students think about their quality of life? A qualitative study. BMC Med Educ..

[26] Bengtsson M (2016). How to plan and perform a qualitative study using content analysis. NursingPlus Open.

[27] Trad LA, Bomfim. Grupos focais: conceitos, procedimentos e reflexões baseadas em experiências com o uso da técnica em pesquisas de saúde. Physis. 2009:777–96 10.1590/S0103-73312009000300013.

[28] Stalmeijer RE, McNaughton N, Van Mook WN (2014). Using focus groups in medical education research: AMEE Guide No. 91. Med Teach.

[29] Tempski P, Santos IS, Mayer FB, Enns SC, Perotta B, Paro HBMS, et al. Relationship among Medical Student Resilience, Educational Environment and Quality of Life. PLoS One. 2015;10(6):e0131535. 10.1371/journal.pone.0131535. 10.1371/journal.pone.0131535 PMC4486187 26121357

[30] Does my study require ethical approval? University of Cambridge, 2020. Available from: https://www.bio.cam.ac.uk/psyres/approval;

[31] Denzin N, Lincoln YS (2003). The Landscape of Qualitative Research: Theories and issues.

[32] Patton MQ (1990). Qualitative Evaluation and Research Methods.

[33] Gerull K, et al. Assessing gender bias in qualitative evaluations of surgical residents. The American Journal of Surgery 217. 2019:306e313. 10.1016/j.amjsurg.2018.09.029. 10.1016/j.amjsurg.2018.09.029 PMC8687875 30343879

[34] Câmara, R. Análise de conteúdo: da teoria à prática em pesquisas sociais aplicadas às organizações. Revista Interinstitucional de Psicologia, 6 (2), jul - dez, 2013,179-191;

[35] A. R. Mozzato, D. Grzybovski. Análise de Conteúdo como Técnica de Análise de Dados Qualitativos no Campo da Administração: Potencial e Desafios. RAC, Curitiba, v. 15, n. 4, pp. 731-747, Jul./Ago. 2011;

[36] Nowell et al. Thematic Analysis: Striving to Meet the Trustworthiness Criteria. International Journal of Qualitative Methods Volume 16: 1–13, 2017. DOI: 10.1177/1609406917733847;

[37] Caregnato RCA, Mutti R. PESQUISA QUALITATIVA: ANÁLISE DE DISCURSO VERSUS ANÁLISE DE CONTEÚDO. Texto Contexto Enferm, Florianópolis, 2006 Out-Dez; 15(4): 679-684;

[38] Bugaj TJ, Schmid C, Koechel A, Stiepak J, Groener JB, Herzog W (2017). Shedding light into the black box: A prospective longitudinal study identifying the CanMEDS roles of final year medical students’ on-ward activities. Med Teach..

[39] Rademakers JJDJM, de Rooy N, Ten Cate OTJ (2007). Senior medical students’ appraisal of CanMEDS competencies. Med Educ..

[40] Sahebi A (2021). Prevalence of anxiety, depression, burnout syndrome, and mental health disorders among healthcare workers during the COVID-19 pandemic: A rapid umbrella review of systematic reviews. J Health Soc Sci..

[41] Donohoe J, O’Rourke M, Hammond S, Stoyanov S, O’Tuathaigh C (2020). Strategies for Enhancing Resilience in Medical Students: a Group Concept Mapping Analysis. Acad Psychiatry..

[42] Weaver R (2011). ‘Part of the team’: professional identity and social exclusivity in medical students. MEDICAL EDUCATION.

[43] Blakey et al. Are medical students socially exclusive? A comparison with economics students. Medical Education 2008: 42: 1088–1091 doi:10.1111/j.1365-2923.2008.03126.x; 10.1111/j.1365-2923.2008.03126.x 18811614

[44] Díaz-Iso A, Eizaguirre A, García-Olalla A. Understanding the Role of Social Interactions in the Development of an Extracurricular University Volunteer Activity in a Developing Country. Int J Environ Res Public Health. 2020;17(12) Available from: 10.3390/ijerph17124422. 10.3390/ijerph17124422 PMC7345550 32575522

[45] Pagnin D, De Queiroz V, Oliveira Filho MA, Gonzalez NV, Salgado AE, Oliveira BC, Lodi CS, Melo RM (2013). Burnout and career choice motivation in medical students. Medical Teacher.

[46] Hafferty, F.W. Beyond Curriculum Reform: Confronting Medicine's Hidden Curriculum. Academic Medicine. Vol. 73, n 4. 1998; 10.1097/00001888-199804000-00013 9580717

[47] Mahase, E. Covid-19: medical students to be employed by NHS. BMJ 2020;368:m1156 doi: 10.1136/bmj.m1156 (Published 20 March 2020); 10.1136/bmj.m1156 32198184

[48] Gi A, et al. Letter to the Editor: The Role of Medical Students in the COVID-19 Pandemic in Portugal. Ordem dos Médicos. 2020; 10.20344/amp.13993;. 10.20344/amp.13993 32378508

[49] Soled D et al. Medical Student Mobilization During A Crisis: Lessons From A COVID-19 Medical Student Response Team. Academic Medicine, 2020. DOI: 10.1097/ACM.0000000000003401 (manuscript ahead-of-print); 10.1097/ACM.0000000000003401 PMC7188031 32282373

[50] Kirkpatrick D, Kirkpatrick J. Evaluating Training Programs: The Four Levels. USA; Berrett-Koehler Publishers; 2006. p. 379.

